# Mapping the glial transcriptome in Huntington’s disease using snRNAseq: selective disruption of glial signatures across brain regions

**DOI:** 10.1186/s40478-024-01871-3

**Published:** 2024-10-21

**Authors:** Sunniva M. K. Bøstrand, Luise A. Seeker, Nadine Bestard-Cuche, Nina-Lydia Kazakou, Sarah Jäkel, Boyd Kenkhuis, Neil C. Henderson, Susanne T. de Bot, Willeke M. C. van Roon-Mom, Josef Priller, Anna Williams

**Affiliations:** 1 Centre for Regenerative Medicine, Institute of Regeneration and Repair, University of Edinburgh, Edinburgh, UK; 2 Institute for Stroke and Dementia Research, Ludwig-Maximilians-Universität, LMU Hospital, Munich, Germany; 3 Department of Human Genetics, Leiden University Medical Center, Leiden, The Netherlands; 4 Centre for Inflammation Research, Institute of Regeneration and Repair, University of Edinburgh, Edinburgh, UK; 5 MRC Human Genetics Unit, Institute of Genetics and Cancer, University of Edinburgh, Edinburgh, UK; 6 Department of Neurology, Leiden University Medical Center, Leiden, The Netherlands; 7 CCBS and UK Dementia Research Institute, University of Edinburgh, Edinburgh, UK; 8 Department of Psychiatry and Psychotherapy, School of Medicine and Health, TU Munich, Munich, Germany; 9 Neuropsychiatry and DZNE, Charité Universitätsmedizin Berlin, Berlin, Germany

**Keywords:** Huntington’s disease, Transcriptomics, Glia, Chaperones

## Abstract

Huntington’s disease (HD) is an autosomal dominant neurodegenerative disease with a fatal outcome. There is accumulating evidence of a prominent role of glia in the pathology of HD, and we investigated this by conducting single nuclear RNA sequencing (snRNAseq) of human post mortem brain in four differentially affected regions; caudate nucleus, frontal cortex, hippocampus and cerebellum. Across 127,205 nuclei from donors with HD and age/sex matched controls, we found heterogeneity of glia which is altered in HD. We describe prominent changes in the abundance of certain subtypes of astrocytes, microglia, oligodendrocyte precursor cells and oligodendrocytes between HD and control samples, and these differences are widespread across brain regions. Furthermore, we highlight possible mechanisms that characterise the glial contribution to HD pathology including depletion of myelinating oligodendrocytes, an oligodendrocyte-specific upregulation of the calmodulin-dependent 3’,5’-cyclic nucleotide phosphodiesterase 1 A (*PDE1A*) and an upregulation of molecular chaperones as a cross-glial signature and a potential adaptive response to the accumulation of mutant huntingtin (mHTT). Our results support the hypothesis that glia have an important role in the pathology of HD, and show that all types of glia are affected in the disease.

**Supplementary Information:**

The online version contains supplementary material available at 10.1186/s40478-024-01871-3.

## Introduction

Huntington’s disease (HD) is a rare, autosomal dominant neurodegenerative disorder characterised by choreatic movements, psychiatric and cognitive disturbances [1]. The disease commonly manifests in midlife [2], and the disease typically progresses over a period of up to 20 years before it becomes fatal. HD is caused by a CAG repeat expansion in the first exon of the *huntingtin (HTT)* gene resulting in an expanded polyglutamine (polyQ) repeat in the huntingtin (HTT) protein. Mutant (m) *HTT* RNA and protein causes cellular toxicity by disrupting a number of processes such as proteostasis, transcription and mitochondrial function and the mutant protein forms aggregates in the nucleus and cytoplasm of both neurons and glia [3]. The presence of mHTT is known to result in prominent neurotoxicity and death of the Medium Spiny Neurons (MSNs) of the striatum. While this MSN loss is considered a core neuropathological feature, all cell types in the body express HTT, and there is accumulating evidence that white matter (WM) deficits and changes to glia typically occur in the disease, including astroglia, microglia and oligodendroglia (oligodendrocytes and oligodendrocyte precursor cells (OPCs)) [4–9]. This pathology disrupts the motor pathways of the basal ganglia, contributing to the choreatic motor symptoms characteristic of HD [10, 11]. Global changes to WM microstructure are common in people with HD, as evident both in the neuropathology and on magnetic resonance imaging (MRI), and the latter has been shown to associate with the core symptoms of the disease including motor deficits, cognitive, and psychiatric problems [12–14]. WM changes seen on MRI are also observed in premanifest HD with several diffusion tensor imaging (DTI) studies demonstrating alterations to WM microstructure at this stage [15–18], suggesting that these represent an early feature of the disease that manifests prior to motor onset. This emphasises glial pathology in HD which may be more complex than simply being secondary to neuronal loss. Evidence of oligodendroglial dysfunction in HD, with an onset prior to overt neuronal death has also been shown in several mouse models of HD [19, 20], and different transgenic animal models have been used to investigate the effect of *mHTT* expression in oligodendroglial populations. Selective expression of a *HTT* fragment under the oligodendroglial *Plp1* promoter gave rise to a similar motor phenotype to the deficits observed in HD, progressive weight loss and premature death [21], with decreased myelin gene expression, thinner myelin sheaths and shortened oligodendrocyte processes. On transplantation of human embryonic stem cell-derived glial progenitor cells (hGPCs) expressing *mHTT* (with the potential to give rise to oligodendrocytes or astrocytes) into myelin-deficient mice, these animals produce delayed and disrupted myelin compared to those transplanted with control hGPCs [22]. Furthermore, transplantation of hGPCs expressing *mHTT* into healthy mice, led to markedly impaired motor learning and performance as early as 12 weeks [23]. Similarly, transgenic mice with astrocyte-specific expression of *mHTT* also show age-dependent motor function deficits and early death [24]. In a reverse approach, the engraftment of healthy hGPCs (biased towards astroglia by sorting for CD44+) in the R6/2 mouse model of HD, prevented reduction in striatal volume, partially rescued cognitive and motor performance and significantly increased survival. Similarly, reduction of *mHTT* expression in mature astrocytes in the BACHD mouse model resulted in a slowing of motor and neuropsychiatric disease progression, as well as rescue of synaptic marker expression and improved neurotransmission in MSNs [25]. In contrast, reducing *mHTT* levels in brain microglia by 50% rescued the inflammatory phenotype in the BACHD mouse model of HD [26], but did not prevent brain volume reduction or improve the phenotype. However, microglia are hyperactive in HD [27] and were recently found to engulf corticostriatal synapses in a complement-dependent manner resulting in cognitive deficits in HD transgenic mice [28]. In light of this accumulating evidence that glia are important in HD, we hypothesised that there are disease-specific glial transcriptome signatures in HD. As glia are heterogeneous across different regions of the CNS [29–31], we also hypothesised that these differences might vary with region. We investigated this using snRNAseq from the post-mortem brains of people with HD and matched controls, and found abundance and gene expression changes in all glial types in the human HD brain compared to controls, in the caudate nucleus (as classically affected in HD) but also more globally in the cerebellum, frontal cortex and hippocampus. We detect depletion of myelin-forming oligodendrocytes, an oligodendrocyte-specific modulation of calmodulin-dependent 3’,5’-cyclic nucleotide phosphodiesterase 1 A (*PDE1A*) expression, which may lead to cellular dysfunction, and an upregulation of chaperone-mediated protein folding as a cross-glial signature potentially acting as a compensatory mechanism. This confirms the importance of glial responses in HD and provides us with possible targets for their manipulation for therapeutic benefit.

## Methods

### Post mortem tissue for snRNAseq

Fresh frozen brain samples from HD and non-neurological control donors were provided by prospective donor schemes with full ethical approval from the MRC Edinburgh Brain Bank (EBB; 16/ES/0084), the Netherlands Brain Bank (NBB; 2009/148), and Leiden University Medical Center (LBB). For tissue from LBB, written informed consent was obtained for each donor in accordance with the Declaration of Helsinki and all material and data were handled in a coded fashion maintaining patient anonymity according to Dutch national ethical guidelines (Code for Proper Secondary Use of Human Tissue, Dutch Federation of Medical Scientific Societies). HD diagnosis was confirmed by neuropathologists and samples assigned a Vonsattel grade. Six HD donors were selected from LBB and NBB, three of each sex. The age range was 48-71y, and four of the six donors received euthanasia. From each donor, four different brain regions were included: Frontal cortex (FrCx), Caudate Nucleus (CN), Hippocampus (HC) and Cerebellum (CB). Control tissue matched for sex and age (range 45-68y) was sourced from all three brain banks and assessed for pathology including Braak staging and amyloid load. See Table 1 for summarised donor information with age, sex and ID of each donor removed to prevent donor identification and more detail in Table S1.

**Table 1:** Summary of donor information. TAAD = Type A dissection of ascending aorta. PMD = Post mortem delay

Brain bank	PMD (h)	CAG length	Diagnosis	Vonsattel grade	Cause of death	
NBB	10:00	na	Non-demented control	na	TAAD	
NBB	05:50	na	Non-demented control	na	Cancer	
NBB	05:30	na	Non-demented control	na	Euthanasia	
NBB	07:30	na	Non-demented control	na	Euthanasia	
Leiden	03:30	na	Non-demented control	na	Myocardial infarct	
EBB	42:00:00	na	Non-demented control	na	Coronary atherosclerosis	
EBB	40:00:00	na	Control - Schizophrenia	na	Suspension by ligature	Excluded
NBB	04:25	?/43	Huntington’s disease	3	Euthanasia	
NBB	06:40	18/42	Huntington’s disease	3	Euthanasia	
NBB	05:40	17/54	Huntington’s disease	3	Euthanasia	
Leiden	04:30	23/43	Huntington’s disease	4	Pneumonia	
Leiden	04:30	20/44	Huntington’s disease	2	General decline	
Leiden	02:00	21/45	Huntington’s disease	3	Euthanasia	

## Nuclear isolation

Depending on tissue size, 3–5 cryosections at 20 μm thickness were used for nuclear extraction for each tissue sample, including both white and grey matter for nuclear preparation. Extraction of nuclei was performed with the Nuclei PURE Prep Isolation Kit (Sigma-Aldrich, NUC201-1KT) as described in https://www.protocols.io/view/nuclei-isolation-from-human-cnssamples-using-nuc2-261genq1og47/v1. Nuclei were stained with Trypan blue, counted using an automated cell counter (Bio-Rad TC20), and normalised to a concentration of 100,000 nuclei per ml.

## Single nuclei RNA sequencing and next generation sequencing

We used the 10X Genomics 3’ gene expression V3 kits and reagents according to the manufacturer’s instructions. Samples were randomised to one of 7 different 10X Genomics Chromium single cell chip B for generation of Gel Bead-in-Emulsion (GEM), with 8000 nuclei loaded per sample. The quality of cDNA libraries was assessed using a Bioanalyzer (PerkinElmer LAS (UK) Ltd), to ensure a minimum library concentration of 5nM. Unique Illumina sequencing primers were added to each sample to allow demultiplexing of sequencing pools. Paired-end next generation sequencing of the cDNA libraries was carried out by Edinburgh Genomics. Indexed libraries were pooled together in four pools of 9 and one pool of 8 libraries. Each pool was sequenced on two lanes of an Illumina NovaSeq S2 flow cell to yield approximately 1750 M read pairs per lane.

## Data preprocessing

FASTQ files were aligned with the human reference genome GRCh38 using 10X Genomics CellRanger v3.0.2. Velocyto (v.0.17.16) [32] was used to reintroduce unspliced mRNAs to the feature count matrix.

## Quality control

Quality control was carried out using Scater [33]. Genes expressed in ≤ 200 nuclei across the spliced and unspliced mRNAs were removed. Nuclei were filtered out based on high/low UMI counts, high/low gene counts and high percentage of mitochondrial genes (see Table S2 for thresholds). Doublets were removed using scDblFinder [34], filtering out nuclei with a doublet score ≥ 0.94. After QC, we show that nuclei quality metrics compared favorably to that seen in previously published data sets (Fig. S1).

### Normalisation and linear dimensional reduction

After QC, the data were normalised using Scran [35] separately for the four tissue regions, then data from the different regions was merged before performing principal component analysis.

## Batch correction

Canonical correlation analysis (CCA) using Seurat v4 [36] was used to adjust for batch effects between different 10X chips. The top 2000 variable genes were selected for each 10X chip, and using the first 37 principal components, we identified integration anchors between each pair of chips, subsequently using these anchors to carry out iterative pairwise integration of batches.

## Dimensional reduction and clustering

Seurat v4 was used for the non-linear dimensional reduction and clustering of cell populations. The 35 first principal components were selected to construct a UMAP.

A K-Nearest Neighbour (KNN) graph was built as basis for community detection using a Louvain algorithm [37]. Initial clustering was performed to resolve broad cell types, as denoted by expression of canonical markers detailed in the main text, using a KNN resolution of 0.25. As an additional measure to reduce the individual differences typical for human samples, we assessed the donor contributions to each cluster. Where one donor contributed more than 50% of a cluster, or a cluster contained nuclei from fewer than 5 donors contributing at least 2% of the total cluster size, or the size of the cluster was very small (fewer than 100 nuclei), the cluster was removed. In addition, one control sample was entirely excluded from further analysis due to a diagnosis of Schizophrenia, which may affect gene regulation in the brain.

### Annotation and subclustering

Subclustering for each lineage was carried out by extracting the relevant broad cluster(s), and applying the same clustering approach as above, selecting the most appropriate number of principal components based on the elbow heuristic. The most appropriate KNN resolution for each cell type was selected after assessing the cluster purity, cohesion and stability at a number of different resolutions. Neuronal populations were annotated with a number and regional specificity where appropriate, and vascular cells according to lineage and function. Glial populations were annotated with the aim to avoid giving an impression of order or rank, and to separate them from previous datasets as follows: Microglia with flower names, astrocytes with herb names, and oligodendroglia with tree names.

### Differential gene expression analysis

Differential gene expression analyses for the identification of cluster marker genes and genes altered in disease were performed using MAST [38], filtering genes for those that had a minimum positive log2-fold change (log2FC) of 0.25 and were expressed by at least 25% of cells within the cluster/group of interest and less than 60% outwith. When comparing HD and control, additional thresholds of absolute log2FC of ≥ 0.8 and adjusted p-value of *<* 0.05 was applied. For differential gene expression analysis of subclusters, oligodendroglia data from the CB were excluded due to the very low number of oligodendroglia captured. The top 5 cluster markers for each cluster of interest were selected by ranking on the log2FC.

### Network and gene ontology analysis

Protein-protein interaction (PPI) and gene ontology (GO) analysis was carried out in Cytoscape (version 3.8.2). We used STRING to build a PPI network of either shared cluster marker genes, or DE genes between HD and control. We then searched for functional enrichment terms associated with the genes in this network, filtering the results to include only terms related to biological processes.

### Differential abundance

Differential abundance (DA) analysis was carried out using MiloR [39]. A KNN graph was constructed and was sampled for downstream analysis using a 0.05–0.1 proportion (depending on cluster size) of sampled cells. The comparison between HD and control was carried out with tissue region as a covariate. Reported p-values are corrected for multiple testing by accounting for overlap between neighbourhoods through neighbour distance weighted FDR correction, using the Vertex method.

### FFPE post mortem tissue for validation

Formalin fixed paraffin embedded (FFPE) tissue from HD donors and age-sex matched controls was obtained with full ethical approval from the MRC Edinburgh Brain Bank (EBB; 16/ES/0084) and the London Neurodegenerative Diseases Brain Bank (LNDBB; 18/WA/0206). These were prepared and delivered as 4 μm sections by the respective brain banks. See Table 2 for list of tissue samples, again with donor ID excluded to ensure these are not identifiable.

**Table 2:** List of human FFPE PM tissue used for in situ hybridisation

Brain Bank	Status	Sex	Age	Vonsattel Grade
LNDBB	Ctrl	F	43	na
LNDBB	Ctrl	M	50	na
LNDBB	Ctrl	F	55	na
EBB	Ctrl	F	71	na
EBB	Ctrl	F	50	na
LNDBB	Ctrl	M	64	na
LNDBB	HD	F	56	3
LNDBB	HD	F	44	2
LNDBB	HD	M	63	3
LNDBB	HD	M	50	3
LNDBB	HD	M	64	4
EBB	HD	F	50	2
EBB	HD	F	74	?

### In situ hybridisation using RNAScope or BaseScope

RNAScope^®^ fluorescent in situ hybridisation (FISH; ACD Bio) was carried out using the RNAScope Multiplex Fluorescent v2 kit (323110) according to manufacturer’s instructions, using the standard pretreatment conditions. Slides were mounted using ProLong Glass (ThermoFisher, P36980). Probes used for RNAScope were Hs-PDE1A (46121), Hs-MBP-C2 (411051-C2), Hs-ITGAM (555091), Hs-BAG3-NoXMmRn-C3 (550871-C3). Secondary dyes used were OPAL-570 (Akoya Biosciences, FP1488001KT) and OPAL-650 (FP1496001KT). BaseScope^®^ in situ hybridisation (ISH; ACD Bio) was carried out using the BaseScope Duplex Reagent kit (323800) according to manufacturer’s instructions. Probes used for BaseScope were BA-HsOLIG2-3zz-st-C1 (1037411-C1) and BA-Hs-PDE1A-3EJ-C2 (1190411-C2). For both staining protocols, two FFPE samples were excluded due to poor staining quality.

### Imaging and quantification

Whole-slide images of RNAScope/BaseScope stained slides were obtained at 40X magnification using a Vectra Polaris slide scanner (Perkin-Elmer/Akyoa Biosciences). Quantification was carried out blinded to the donor ID and disease status of the sample. Image quantification was carried out in QuPath v0.2.3 [40]. Regions of interest (ROIs) were defined as 500µm x 500µm squares, and for each of the 12 tissue Sect. 8 ROIs were selected. For BaseScope, this was not always possible due to variability in the signal, and in those cases we selected as many ROIs with detected signal as possible. For RNAScope, DAPI-positive nuclei were identified using manual thresholds, and for each nucleus and a 1µm perimeter around it, positive mRNA molecules were detected automatically using the ”subcellular detection” function in QuPath. For BaseScope, quantification of single and double positive cells was carried out manually. We then calculated the double positive cells expressed as a proportion of all cells expressing the relevant lineage marker. Statistical analysis was carried out using this proportion as the outcome variable. Image quantification data is presented as boxplots showing median value and range. To compare groups, a Welch Two Sample t-test was used for data with normal distribution and a Wilcoxon rank sum test for non-normally distributed data. The Shapiro Wilk test was used to assess normality of data.

## Results

### snRNAseq of four regions of PM brain from HD cases and controls

**Fig. 1:**
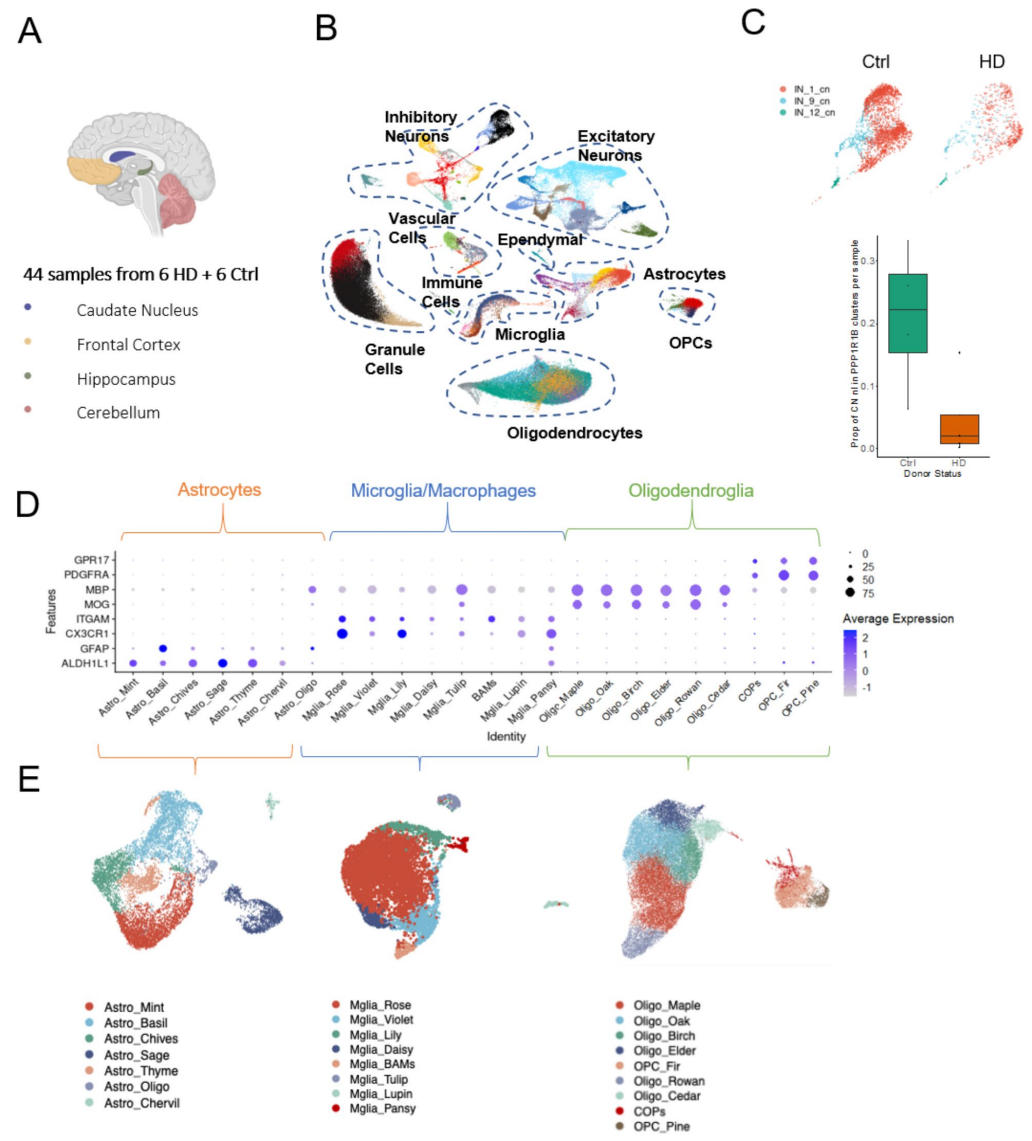
snRNAseq of four regions of human post mortem brain from HD donors and controls. **A**. Sample summary. **B**. UMAP plot of all nuclei across brain regions, disease and control, showing all major cell types in the brain. **C**. The proportion of total nuclei identified as medium spiny neurons in the CN (PPP1R1B+) is reduced in HD compared to control (plot shows median and range). **D** Expression of canonical markers for cell lineages, across glial populations. **E** UMAP plots of astrocytes, microglia and oligodendroglia (L-R), showing annotated subclusters. BAM = border-associated macrophages

We were interested in investigating the glial signatures associated with HD both in areas of MSN loss (caudate nucleus) and beyond, so we carried out snRNAseq of four regions of PM brain: caudate nucleus (CN), cerebellum (CB), frontal cortex (FrCx), and hippocampus (HC) from 6 HD cases, and 6 age and sex matched controls (Fig. 1A; Table 1). Following quality control of cDNA libraries, nuclei, samples and clusters, our dataset included 127,205 nuclei across 44 tissue samples, and compared favourably with quality and number from previous published datasets [29, 41, 42] (Fig. S1). We first interrogated the expression of canonical markers to identify all the major cell types of the brain (Fig. 1B). We confirmed the presence of astrocytes (*ALDH1L1*,* GFAP*), microglia (*CX3CR1*,* ITGAM*), oligodendrocytes (*MOG*,* MBP*), OPCs and committed OPCs (COPs; *PDGFRA*,* GPR17*), endothelial cells (*VWF*,* CLDN5*), pericytes (*PDGFRB*,* NOTCH3*), peripheral immune cells (*CD2*,* CD8*), ependymal cells (*CFAP299*,* SOX9*), excitatory neurons (*SLC17A7*), inhibitory neurons (*GAD2*) and cerebellar granule cells (*RELN*,* SLC17A7*). Within the inhibitory neuron population, we identified three clusters in the CN expressing the medium spiny neuron marker *PPP1R1B*, and we found that in HD the proportion of median spiny neurons per all CN nuclei was reduced (Fig. 1C), reflecting the hallmark pathology of the disease. As the role of glia in HD pathology is less well understood than that of neurons, and the number of differentially expressed (DE) genes comparing HD and control was higher in glia than in neurons (313 DE genes across neuronal subclusters and regions, 1278 across glia), we focussed on the microglia, astrocytes, and oligodendroglia to provide a comprehensive characterisation of glial changes in HD. We subsetted our data for the clusters expressing high levels of canonical glia markers: microglia (*CX3CR1*,* ITGAM*), astrocytes (*ALDH1L1*,* GFAP*), and oligodendroglia (oligodendrocytes: *MOG*,* MBP*, OPCs: *PDGFRA*, COPs: *GPR17*), and reclustered the nuclei within these subsets. We identified 9 clusters of oligodendroglia (6 oligodendrocytes (Oligo), 2 OPCs, 1 COPs), 8 subclusters of microglia (Mglia), and 7 subclusters of astrocytes (Astro; Fig. 1D-E).

### Altered glial abundance in HD

**Fig. 2:**
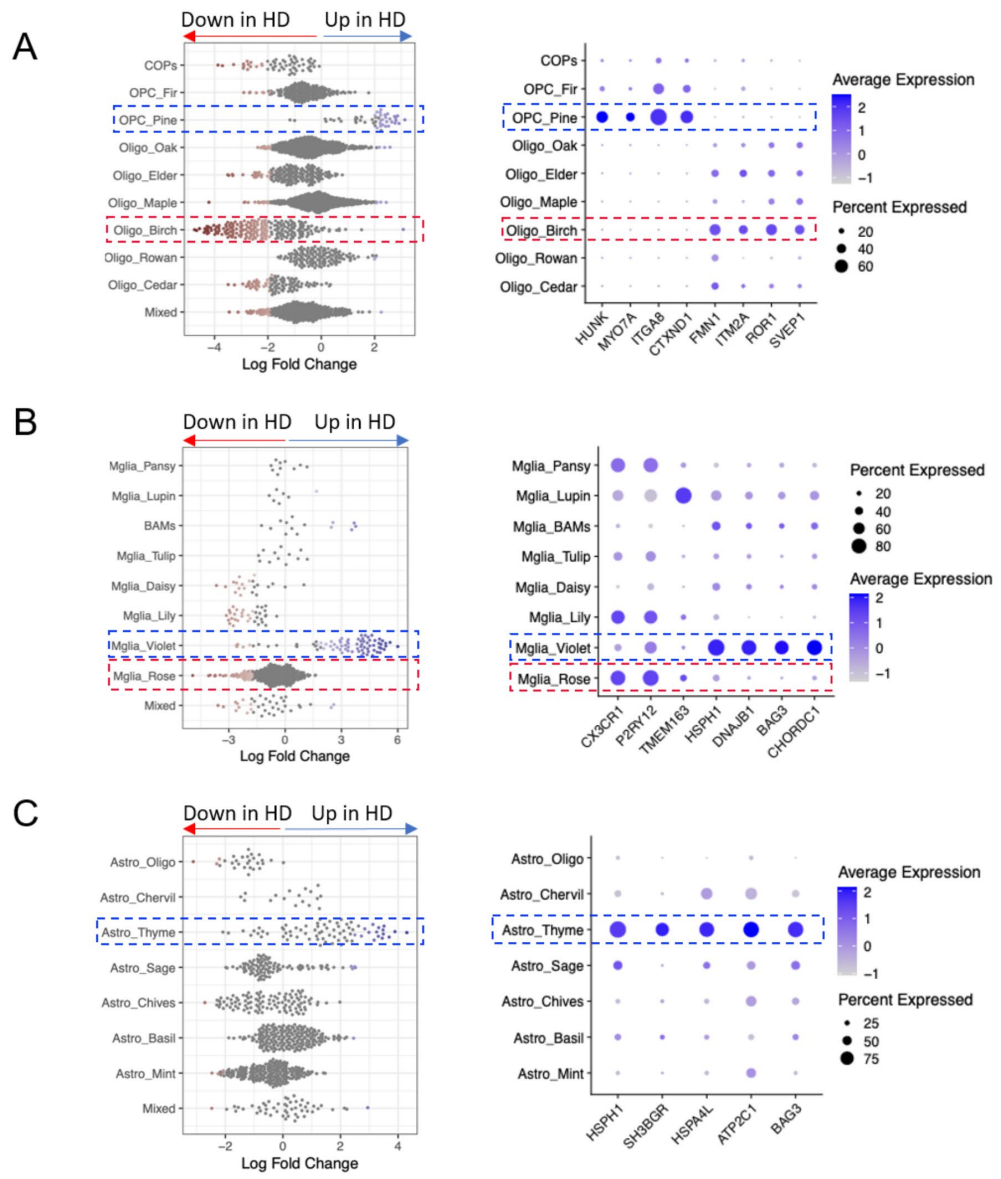
Altered glial abundance in HD. **A-C** (left). Beeswarm plots showing differential abundance of subclusters of oligodendroglia (**A**), microglia (**B**), astrocytes (**C**), with markers of clusters that were significantly enriched (highlighted in blue) or depleted (highlighted in red) in HD compared to controls. **A-C** (right) Corresponding dot plots showing selected marker genes for the glial subclusters

To investigate changes in the relative proportions of the identified glial subclusters between HD and control donors, we carried out differential abundance analysis across the four brain regions combined. The results showed changes in the abundance of several glial subpopulations, with some clusters enriched and others depleted (Fig. 2A-C). Most prominently, we observed a depletion of Oligo Birch (marked by *FMN1*,* ITM2A*,* ROR1*,* SVEP1*, Fig. 2A), Mglia Rose (marked by *CX3CR1*,* P2RY12*,* TMEM163*, Fig. 2B), and an enrichment of OPC Pine (marked by *HUNK*,* MYO7A*,* ITGA8*,* CTXND1* Fig. 2A), Mglia Violet (marked by *HSPH1*,* DNAJB1*,* BAG3*,* CHORDC1*, Fig. 2B) and Astro Thyme (marked by *HSPH1*,* SH3BGR*,* HSPA4L*,* ATP2C1*,* BAG3*, Fig. 2C). We assessed whether this varied by tissue region and found most prominent depletion of Oligo Birch in HD in the CN and most prominent enrichment of Astro Thyme in HD in the HC, OPC Pine in the CB and HC, but Mglia Violet showed widespread increase in all four regions (Fig S2). Astrocytes showed a strong regional profile independent of donor status with Astro Sage being highly predominant in the CB, expressing the markers *PAX3*,* NTNG2* and *ATP10B* similar to that found in a CB-specific type of astrocyte already described [29] (Fig S2).

### Oligodendroglia show selective depletion and altered gene expression

**Fig. 3:**
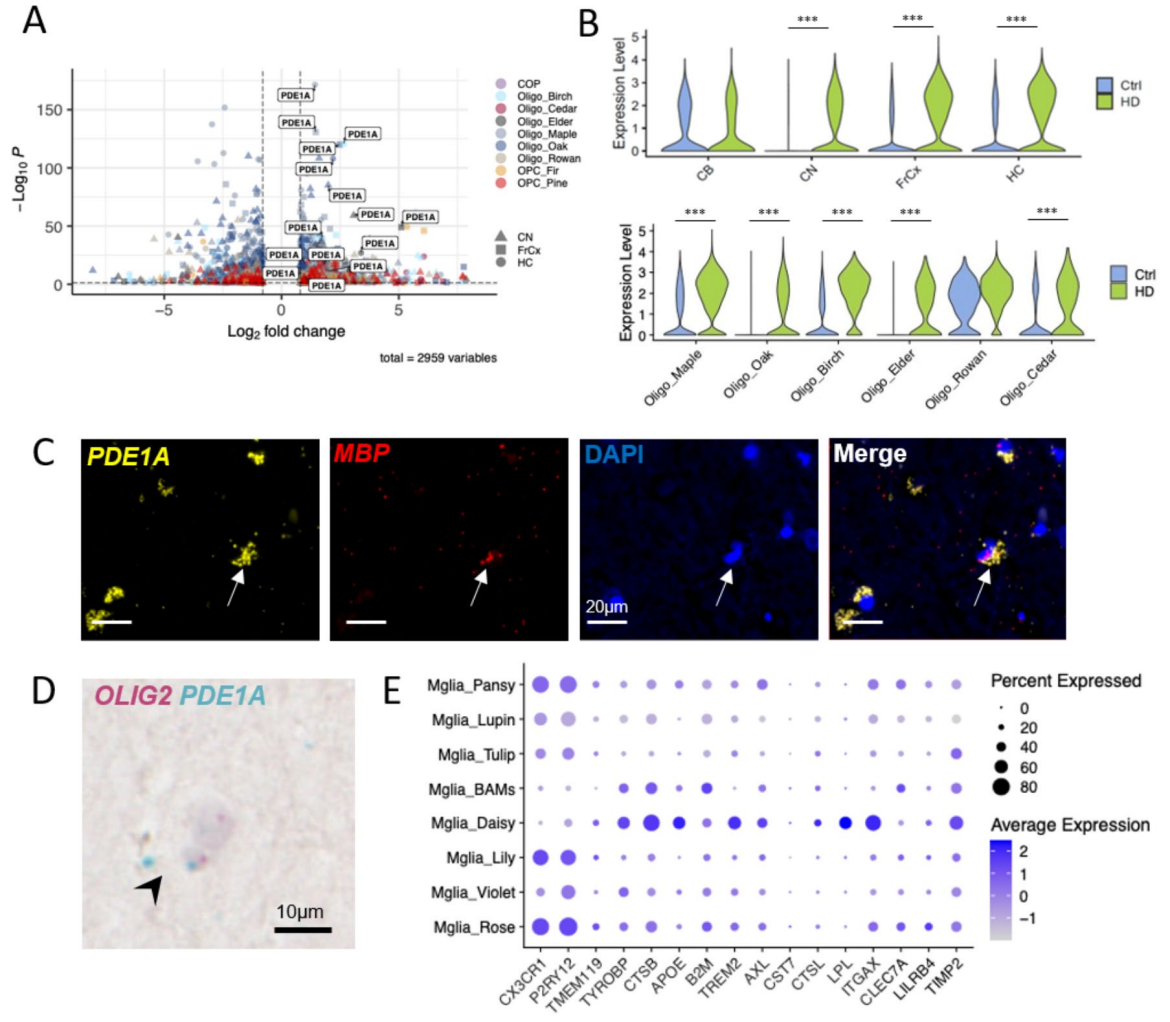
*PDE1A *upregulation in oligodendrocytes in HD **(A)** We identified upregulation of *PDE1A* across multiple clusters and regions. **(B)** Violin plots of expression of *PDE1A* in different regions and different clusters comparing HD and control donors. Stars represent adjusted p-values from MAST, *** p *<* 0.001. **(C)** Duplex RNAScope identification of *PDE1A* transcript (yellow) in MBP-expressing (red) oligodendroglia in human HD brain. DAPI-stained nuclei in blue. **(D)** BaseScope identification of *PDE1A* transcript (blue) in *OLIG2* transcript-expressing (pink) oligodendroglia in human HD brain. **(E)** Dot plot showing expression of immune markers across microglial subclusters

We investigated the severely depleted oligodendrocyte cluster Oligo Birch, and found that this expressed *OPALIN*, previously described as a marker of newly formed oligodendrocytes [43] and myelin-forming oligodendrocytes in rodents [44], as well as the cytoskeletal component *FMN1*. The expression of these two genes in this cluster suggests Oligo Birch as an actively myelinating population of oligodendrocytes, and the loss of this cluster of myelin forming oligodendrocytes is consistent with the myelin changes seen in HD and preclinical HD models [6, 20, 45]. Differential gene expression (DGE) analysis showed upregulation of the gene encoding cyclic nucleotide phosphodiesterase *PDE1A* in multiple oligodendrocyte clusters in HD in all brain regions examined except cerebellum and in five out of the six oligodendrocyte clusters (Fig. 3A, B). We confirmed expression of *PDE1A* transcript in MBP-expressing oligodendroglia in a separate set of FFPE samples from PM brain of HD donors by RNAScope (Fig. 3C). We also used BaseScope to confirm the expression of *PDE1A* transcript in OLIG2-expressing oligodendroglia (Fig. 3D). PDE1A belongs to a large group of cyclic nucleotide phosphodiesterase families that hydrolyze cGMP and/or cAMP to their respective monophosphates, with unique calmodulin-dependent activation and a higher affinity for cGMP.

### Microglia are activated in HD

Another prominent change was the depletion of Mglia Rose, marked by *P2YRY12*,* CX3CR1*,* TMEM163*, identifying this as a more homeostatic population of microglia (Fig. 3E) and an increase in Mglia Violet, showing more markers of metabolic activation. This suggests that a subset of microglia transitions from a homeostatic to an immune-activated phenotype as already described in HD [5]. As disease-associated microglia (DAM) states have been described in rodent models of neurodegenerative disease and ageing [46], we investigated the expression of selected immune genes across all microglia clusters (Fig. 3E), and found that the microglial cluster Mglia Daisy expressed low levels of *CX3CR1*,* P2RY12*,* TMEM119* and high levels of the immune related genes *TYROBP*,* CTSB*,* APOE*,* TREM2*,* AXL*,* CTSL*,* LPL*,* ITGAX*,* TIMP2*. Although we saw an increase in number of more activated microglia in HD, we did not find a microglial upregulation in the expression of the complement genes *C1QA/C1QB/C1QC/C3* in HD to correlate with the increased C1q and C3 protein previously identified in the HD caudate associated with loss of corticostriatal synapses [28].

#### Glial subpopulations enriched in HD upregulate their expression of molecular chaperone genes

**Fig. 4:**
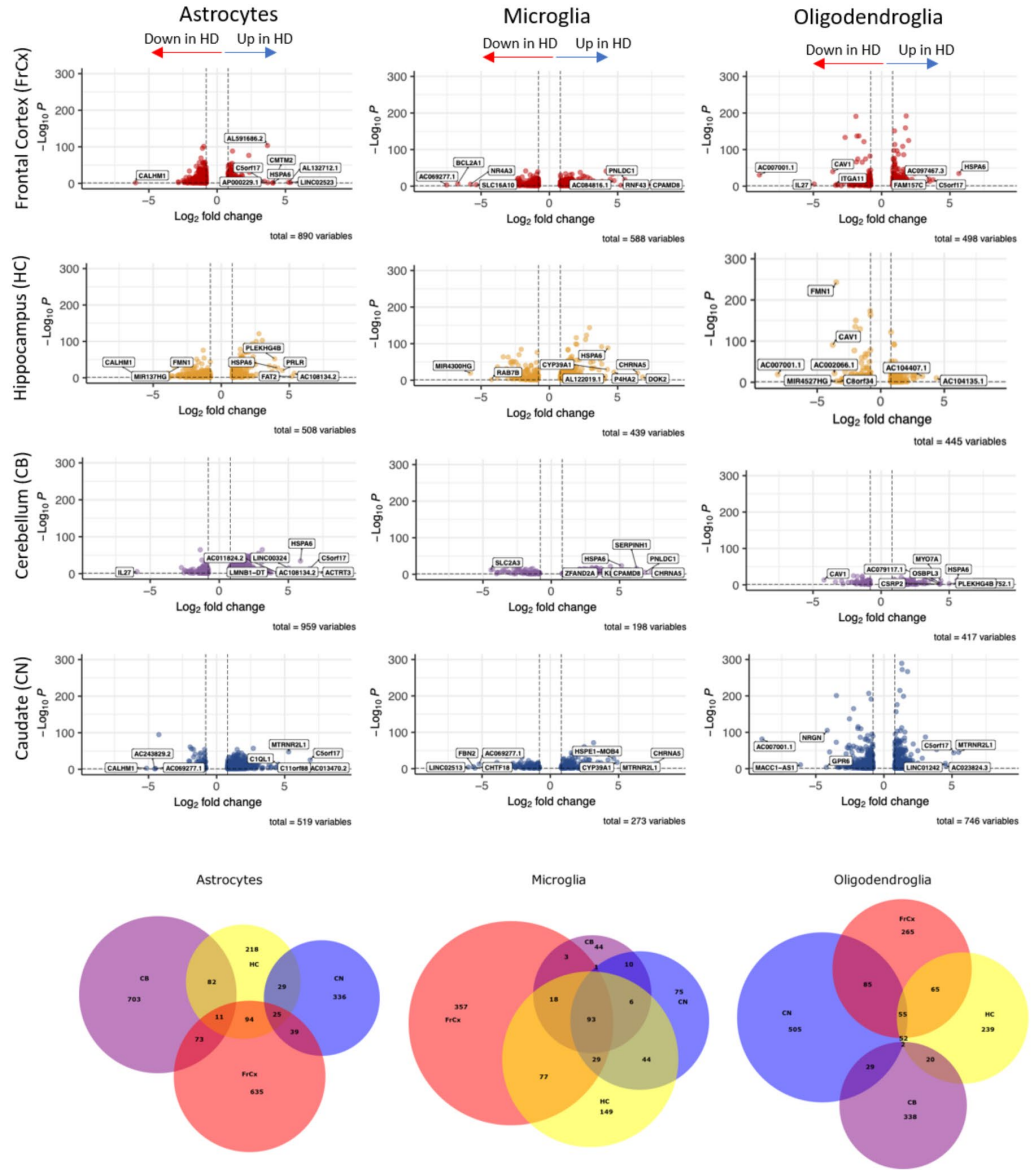
Differential expression analysis between HD and control donors for each glial type and region. Volcano plots showing differentially expressed genes between HD and controls in each glial type and each region. The top eight most differentially expressed genes in either direction are labelled (all in Tables S3-5). Venn diagrams show overlapping and differentially expressed genes between HD and controls per cell type and region

We next compared gene expression differences and similarities in glia from each of the four regions of interest between HD and control donors (Fig. 4 and Tables S3-5). We carried out gene ontology (GO) analysis based on the differentially expressed genes between HD and control for astrocytes, microglia and oligodendroglia in the four tissue regions (top five pathways listed in Fig S3, with full data in Tables S8-10). This analysis showed that these differentially expressed genes between HD and control shared many gene ontology groupings between regions, suggesting that the processes/pathways were rather similar, but with some difference in genes from these pathways identified regionally. There are some regional differences, for example, astrocytes in the CB express more genes related to protein folding, whereas those differentially expressed in the CN are more related to adhesion and cytoskeleton. In microglia, many of the genes differentially expressed between HD and controls are related to chaperone proteins and protein folding in three of the four regions, suggesting regional nuances, but immune activation seems more prominent in the HC microglia. For oligodendroglia, transcripts related to synapses are differentially expressed in HD in all regions except in the CB, and this may reflect the relative loss of mature oligodendrocytes and relative gain of OPCs, which are known to express genes classically considered as synaptic. The similarity of the pathways altered in glia in HD across regions, with the predominance of the chaperone/protein folding signatures, led us to investigate overlapping pathways in more depth.

**Fig. 5:**
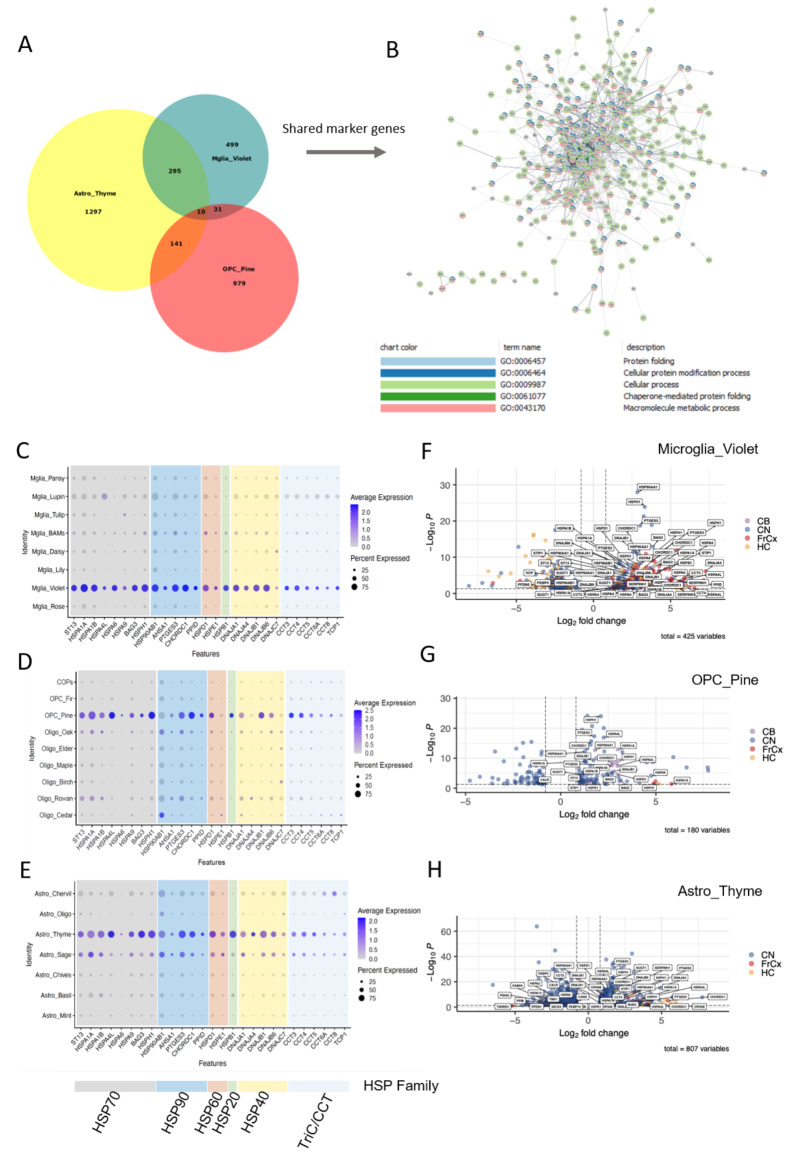
Molecular chaperone genes are upregulated across glial clusters in HD. **A**. Venn Diagram showing overlap of cluster markers between Astro_Thyme, Mglia_Violet and OPC_Pine. **B**. PPI network of shared cluster markers, coloured to denote genes belonging to one of the top 5 GO terms shown below. **C-E.** Expression of shared molecular chaperone genes in microglia, marking Mglia Violet (**C**), oligodendroglia, marking OPC Pine (**D**) and in astrocytes, marking Astro Thyme (**E**). Dot plots are colour coded according to chaperone family (key below). **F-H.** Volcano plots showing differentially expressed genes in Mglia Violet (**F**), OPC Pine (**G**) and Astro Thyme (**H**), labelling altered molecular chaperone genes and regions. Log2FC/p-values from MAST comparing HD and control within each cluster

We analysed shared gene expression in the glial subpopulations enriched in HD (OPC Pine, Astro Thyme, and Mglia Violet) and found many that were overlapping, with 295 shared between Astro Thyme and Mglia Violet, 141 shared between Astro Thyme and OPC Pine and 41 between Mglia Violet and OPC Pine, supporting shared mechanisms across the three glial subtypes (Fig. 5A). To investigate shared pathways, we then constructed a protein-protein interaction (PPI) network for these genes and carried out functional enrichment (Fig. 5B, Table S6). The most significant GO terms included ”protein folding” and ”Chaperone-mediated protein folding”. Shared overlap genes included various molecular chaperones, including members and co-factors of the Heat Shock Protein (HSP) 70 family: *HSPA1A*, *HSPA1B*, *HSPA9*, *BAG3*, *HSPA4*, *HSPA4L*, *HSPH1* and *ST13*, as well as those associated with the HSP90 family: *CHORDC1*, *PPID, HSP90AB1* and *PTGES3*, the small HSP (sHsp) family: *HSPB1*, the HSP40 family: *DNAJB1*,* DNAJB6*,* DNAJA1*, the HSP60 family of chaperonins: *HSPD1*,* HSPE1*, and members of the TriC/CCT complex *CCT3*,* CCT4*,* TCP1* (Fig. 5C-E). These gene alterations represent all major families of molecular chaperones, and are present in all regions, and we note that more members of the HSP70-family are affected, as visualised by plotting expression of the shared chaperone genes for the subclusters of each glial type (Fig. 5C-E). Similarly, differential gene expression (DGE) analysis comparing HD and controls for the three subclusters of interest separately (Mglia Violet, OPC Pine and Astro Thyme), with more stringent thresholds (absolute log2FC > 0.8, FDR-corrected p *<* 0.05), showed widespread changes in expression of the chaperone family genes (Fig. 5F-H).

**Fig. 6:**
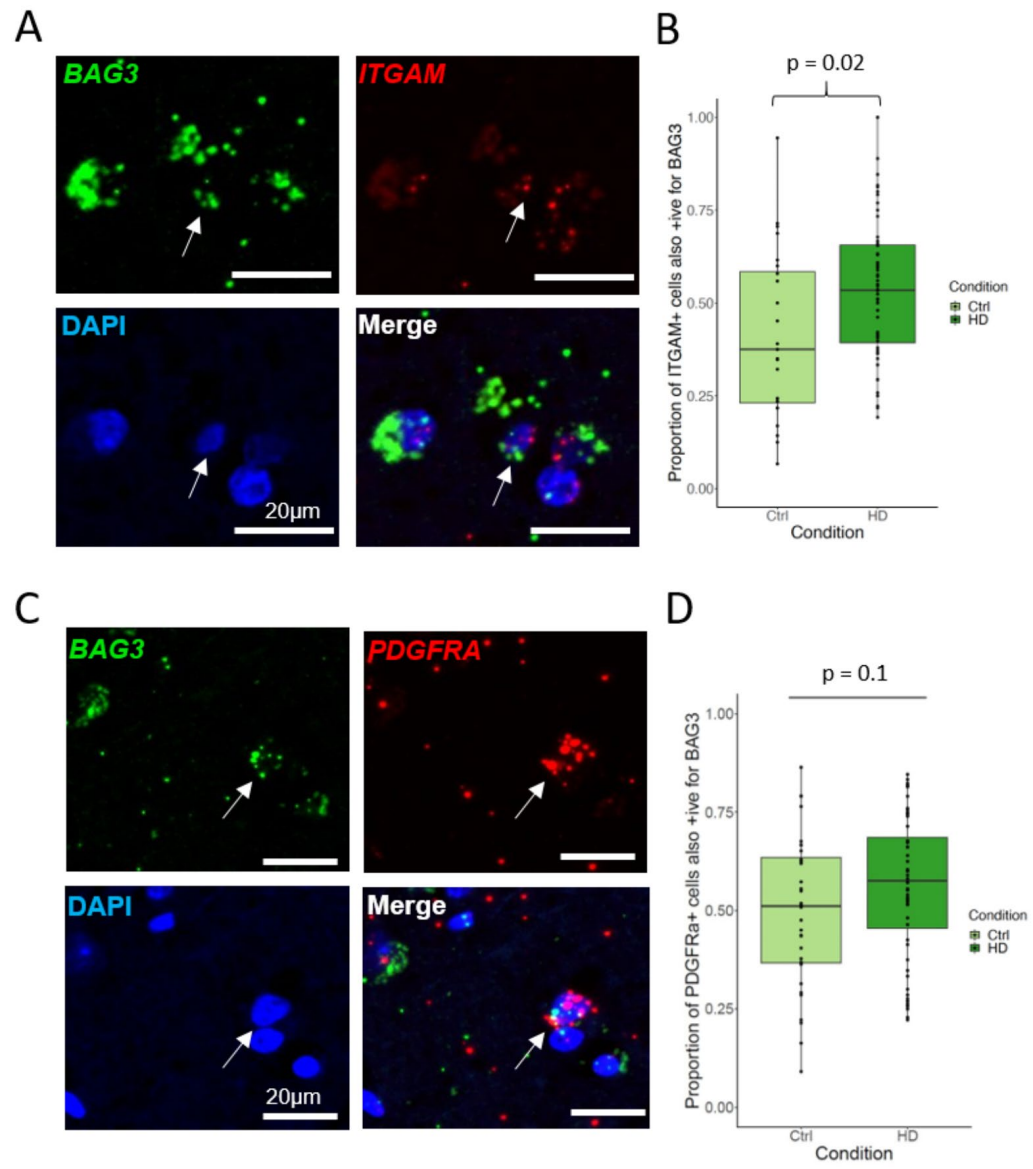
The molecular co-chaperone BAG3 is upregulated in HD microglia in a separate cohort. **A-B**. Double in situ hybridisation (**A**) on a separate human brain tissue cohort showing an increase (**B**) in microglia (*ITGAM*+, red) expressing *BAG3*, green. Arrow for double-positive cell. Plot shows median and range. (Welch Two Sample t-test, p-value = 0.023). **C-D**. Double in situ hybridisation (**C**) showing OPCs (*PDGFRa*+, red) expressing *BAG3*, green (**D**). Plot shows median and range (Wilcoxon rank sum test, p-value = 0.1). DAPI-stained nuclei coloured blue in images

As validation, in a separate set of FFPE samples from PM brain of HD donors and controls (see methods), we combined fluorescent RNAScope in situ hybridisation probes (detailed in methods) for the shared upregulated HSP70 co-chaperone *BAG3* with *ITGAM*, labelling microglia (Fig. 6A, B) or *PDGFRA*, labelling OPCs (Fig. 6C, D). There was an increase in *ITGAM + BAG3 +* microglia in HD versus control (Welch Two Sample t-test: t = -2.3665, df **=** 38.767, p-value = 0.023). Thus, both combined and separately in the more abundant clusters, and over different regions, we confirm a broad cross-glial profile of upregulation of genes involved in protein folding and molecular chaperones affecting all major chaperone families.

## Discussion

The present study represents a comprehensive characterisation of glial changes across different regions of the brain in HD. The results support our hypothesis that the transcriptomic profiles of glia are altered in HD across brain regions, not limited to one type of glial cell but across microglia, astroglia, and oligodendroglia. This was true both in terms of the relative abundance of certain glial subtypes, and their gene expression profiles. The changes in glia in HD are not limited to the striatum, where there is prominent loss of MSNs, but present in all of the areas we examined to varying degrees, confirming that HD has global brain effects. In HD, we found a widespread reduction in actively myelinating oligodendrocytes (Oligo Birch), consistent with the known white matter myelin deficit in HD and in preclinical HD models [6]. Human oligodendrocyte/glial precursor cells containing *mHTT* in vitro [22] or OPCs in preclinical HD models (e.g. BACHD [20] or R6/2 mice [23]) show reduced capacity to differentiate into myelinating oligodendrocytes, both functionally and with reduction in myelin gene transcripts. However, preclinical mouse HD models do not report an overall change in OPC or oligodendrocyte numbers, even at later timepoints [20]. Our data suggest a loss of oligodendrocytes plus a block in ability to restore numbers by differentiation from OPCs, with increased OPCs and reduced myelinating oligodendrocytes. This process is often suggested is a cause of remyelination failure in the classical demyelinating disease multiple sclerosis (MS) [47], but the mode of differentiation block appears different as signatures similar to actively myelinating oligodendrocytes appear more prevalent in MS snRNAseq databases [41, 48]. As well as differences in oligodendroglial numbers, we found an increase in expression of the phosphodiesterase gene *PDE1A* in oligodendrocytes in HD in all regions. Phosphodiesterase family members are involved in oligodendrocyte biology, in that inhibition of PDE7 [49], PDE4 [50] and PDE3 [51] in rodents in vivo and in vitro, increased oligodendroglial differentiation, survival, and remyelination after demyelination. In addition, inhibition of PDE10A improved striatal pathology and behavioural performance in animal models of HD [52, 53], leading to a clinical trial of a PDE10A inhibitor in HD, which was unfortunately unsuccessful [54]. In the heart, *PDE1A* acts through regulation of cGMP levels [55], and PDE1 inhibition improved proteasomal degradation of a misfolded protein in a mouse model of proteinopathy-induced cardiac failure [56], but we do not yet know whether this may be similarly useful in HD models. We highlight upregulation of transcripts related to chaperone-mediated protein folding across glial subsets in HD. Molecular chaperones are well known to be important in neurodegeneration [57] as they play an important role in maintaining proteostasis both in healthy cells where they are constitutively expressed and in the context of disease where they are inducible by stress, disease or injury. The observed upregulation is not likely to be related to post mortem cellular stress, since all examined nuclei passed quality control, the expression changes were selective for some cellular clusters only and not found in the control donors undergoing the same processing. As aggregation of mutant HTT is implicated in HD pathology, the upregulation of chaperone-mediated protein-folding machinery may reflect an adaptive survival response. However, the beneficial or detrimental function of different types of molecular chaperones in HD is complex and the balance between different types may be key. We noted upregulation of more components of the HSP70 family, and in mouse, fly and yeast models, overexpression of HSP70 has been shown to suppress polyQ-aggregation and subsequent neurodegeneration [57, 58]. However, inhibition of HSP90 in these systems also reduces protein aggregation and toxicity [57]. The trimeric chaperone complex, composed of the class II J-domain protein HSP40 family member DNAJB1, Hsc70 and the nucleotide exchange factor Apg2 also targets HTT, with DNAJB1 as the rate-limiting step, directly binding HTT and delaying aggregation and encouraging disaggregation in vitro [59]. The overexpression of the similar protein DNAJB6 in astrocytes was sufficient to delay neurodegeneration in a Drosophila model with neuronal *mHTT* overexpression [60], showing its therapeutic potential. HSP-genes have been previously linked with HD [61], but also to other neurodegenerative diseases such as vascular dementia [62], Parkinson’s disease [63] and multiple sclerosis [48] reflecting the relevance of this system to neurodegeneration. Consistent with other studies [64, 65], we identified that microglia lose their homeostatic signature in HD, and gain other immune activation markers throughout our regions of interest, but also that BAG3-expressing microglia are enriched in HD, of interest as BAG3 expression has recently been linked to AD and PD pathogenesis [66, 67]. The transcriptomic heterogeneity of glia has also been demonstrated in other neurological and psychiatric disorders, including multiple sclerosis [41], Alzheimer’s [42], Parkinson’s [68], and major depressive disorder [69], suggesting a dynamic ability of the cells to change their transcriptomic ”state” in response to disease or injury. Our finding of a cross-glial signature of upregulated chaperone-mediated protein folding provides some evidence of the latter as this likely represents an adaptive response to the presence of *mHTT* in HD. An upregulation of molecular chaperones has previously been reported in HD astrocytes in the cingulate cortex [70] and recently in human HD OPCs and oligodendrocytes [61]. As these are known to aid protein folding, prevent protein aggregation [71] and promote degradation of misfolded proteins by the proteasome [57] in the context of stress/disease/injury, we propose that their upregulation is a potentially beneficial compensatory disease mechanism shared across a subset of OPCs, microglia and astrocytes in HD.

These findings of global transcriptomic changes in glia in HD support a therapeutic disease-modifying strategy with growing support in several neurodegenerative diseases that targeting a pathological mechanism within a glial population may be beneficial. Therefore, our study highlights an opportunity for novel therapeutics in HD.

## Conclusion

In this study, we investigated the transcriptomic profiles of glia in HD in order to understand how these are affected in the disease, which has been traditionally considered neuronal. We found prominent changes in microglia, astrocytes, and oligodendroglia subtypes, both in terms of their relative abundance and gene expression. Notably, these changes were not confined to the caudate nucleus, seen to be most affected in HD. Instead, we find global changes to the glial transcriptome, with some regional nuances. Among the most salient changes in HD gene expression, we found an upregulation of PDE1A selectively in oligodendroglia, across all forebrain regions. We also describe cross-glial perturbation of the molecular chaperones, which may reflect an adaptive response to mHTT accumulation, as these molecules have been shown to counteract mHTT aggregation in experimental models. Our work adds to a growing body of literature demonstrating that glia are an important part of the pathophysiology of HD. Furthermore, as glia have regenerative potential and are therefore more tractable targets for therapy, our findings are of relevance for future disease-modifying therapies targeting glial pathology in HD.

## Electronic supplementary material

Below is the link to the electronic supplementary material.

Supplementary Material 1: Figure S1- Comparison of nuclei quality with two published datasets.

Supplementary Material 2: Figure S2- Cluster proportions comparing regions and conditions.

Supplementary Material 3: Figure S3- Tables of top 5 GO terms comparing genes that are different between HD and controls for the different regions for the different cell types.

Supplementary Material 4: Table S1- Additional donor information, Table S2 - Thresholds used for QC of individual nuclei separated by splice status, Table S3 - List of differentially expressed genes by region in HD microglia, Table S4 - List of differentially expressed genes by region in HD oligodendroglia, Table S5 - List of differentially expressed genes by region in HD astrocytes, Table S6 - List of differentially expressed genes by region in HD oligodendroglia subclusters, Table S7 - STRING enrichment for cluster markers shared pairwise between Astro Thyme, OPC Pine, Mglia Violet, Table S8 - STRING enrichment for DE genes by region in HD microglia, Table S9 - STRING enrichment for DE genes by region in HD oligodendroglia, Table S10 - STRING enrichment for DE genes by region in HD astrocytes

## Data Availability

Data are provided within the manuscript or supplementary information files. Code is publicly available on Github https://github.com/Anna-Williams/ANC_supplementary_repo. RNAseq raw data is available on GEO, referenced in our shiny app available for easy data exploration (https://smkboestrand.shinyapps.io/transcriptomic_profiling_glia_HD/).

## Abbreviations

AD: Alzheimer’s disease
BAM: Border associated macrophage
CAG: Cytosine adenosine guanine
CaM: Calmodulin
cAMP: Cyclic adenosine monophosphate
CB: Cerebellum
CCA: Canonical correlation analysis
cGMP: Cyclic guanosine monophosphate
CN: Caudate nucleus
CNS: Central nervous system
COP: Committed oligodendrocyte precursor cell
Cpn: Chaperonin
DA: Differential abundance
DAM: Disease associated microglia
DE: Differential expression
DEG: Differentially expressed gene
DTI: Diffusion tensor imaging
EBB: Edinburgh Brain Bank
EC: Endothelial cell
EN: Excitatory neuron
FFPE: Formalin fixed paraffin embedded
FISH: Fluorescent in situ hybridisation
FrCx: Frontal cortex
GM: Grey matter
GO: Gene onotology
HC: Hippocampus
HD: Huntington’s disease
hGPCs: Human glial progenitor cells
HSP: Heat shock protein
HTT: Huntingtin
IN: Inhibitory neuron
ISH: In situ hybridisation
KNN: K-nearest neighbour
LBB: Leiden University Brain Bank
LNDBB: London Neurodegenerative Diseases Brain Bank
Log2FC: Log2-fold change
mHTT: Mutant huntingtin
MRI: Magnetic resonance imaging
mRNA: Messenger RNA
MS: Multiple sclerosis
MSN: Medium spiny neuron
NBB: Netherlands Brain Bank
NGS: Next generation sequencing
OPC: Oligodendrocyte precursor cell
PD: Parkinson’s disease
PDE1A: 3’,5’-cyclic nucleotide phosphodiesterase 1A
PM: Post mortem
PMD: Post mortem delay
PPI: Protein-protein interaction network
QC: Quality control
qRT-PCR: Quantitative RT-PCR
ROI: Region of interest
scRNAseq: Single cell RNA sequencing
sHSP: Small heat shock protein
snRNAseq: Single nuclear RNA sequencing
UMAP: Uniform approximation and projection
UMI: Unique molecular identifier
WM: White matter
