# Supplementary figures and images for: Mapping the glial transcriptome in Huntington’s disease using snRNAseq: selective disruption of glial signatures across brain regions
Source: Acta Neuropathol Commun. 2024 Oct 21;12:165. doi: 10.1186/s40478-024-01871-3 (PMC11492505; doi:10.1186/s40478-024-01871-3)

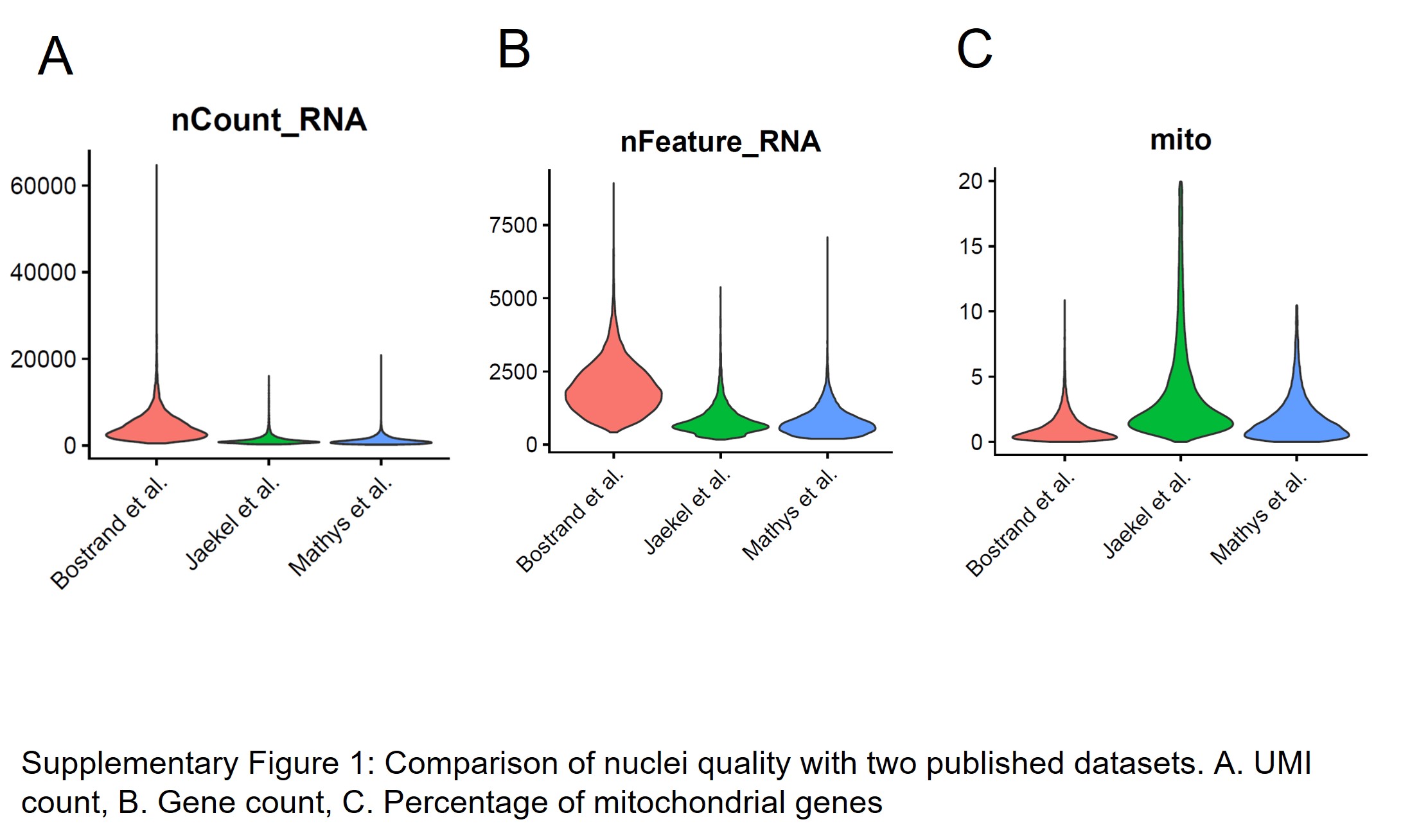

Supplement: Supplementary file 1 — Supplementary Material 1: Figure S1- Comparison of nuclei quality with two published datasets. [file 40478_2024_1871_MOESM1_ESM.jpg]

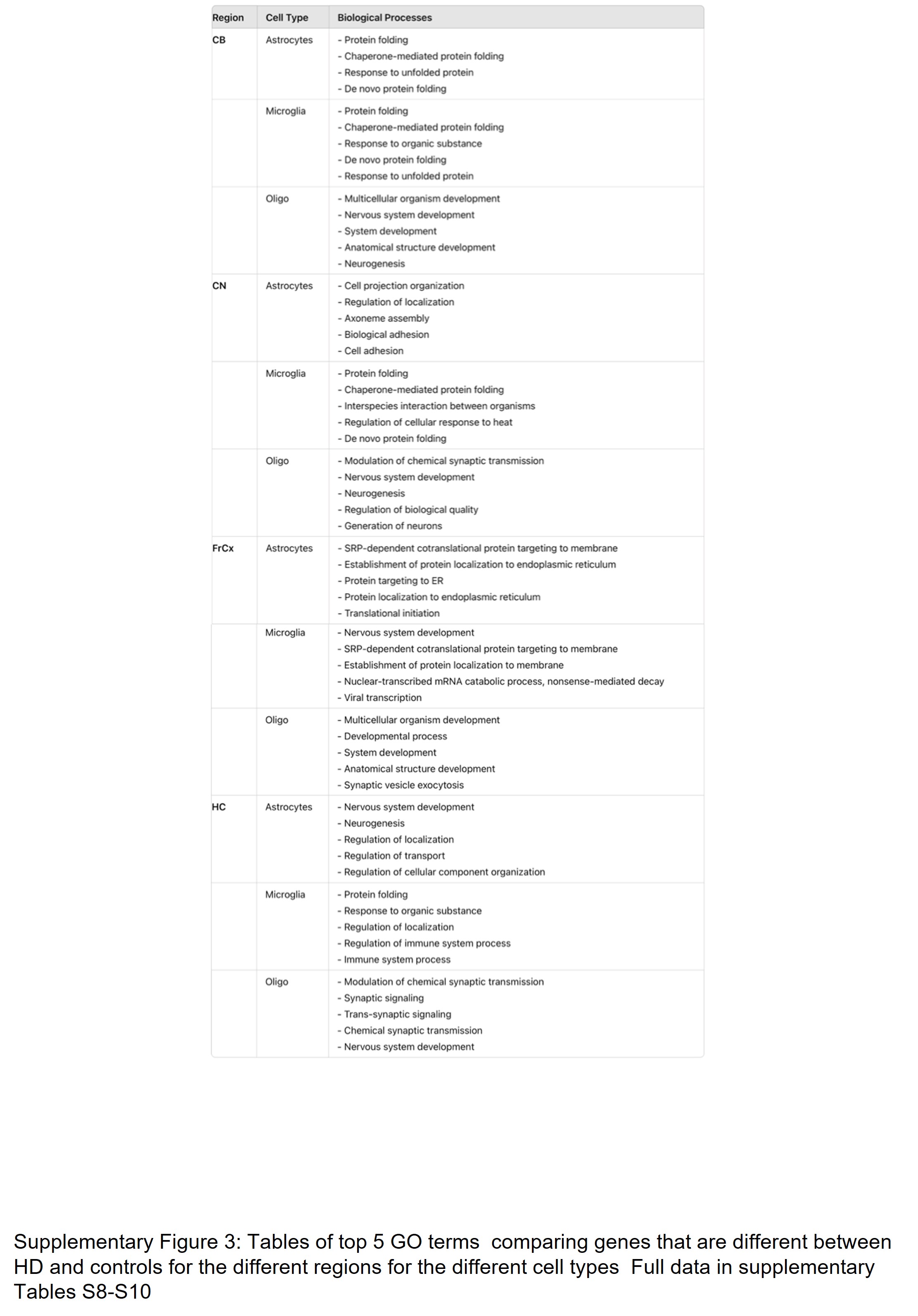

Supplement: Supplementary file 2 — Supplementary Material 2: Figure S2- Cluster proportions comparing regions and conditions. [file 40478_2024_1871_MOESM2_ESM.jpg]

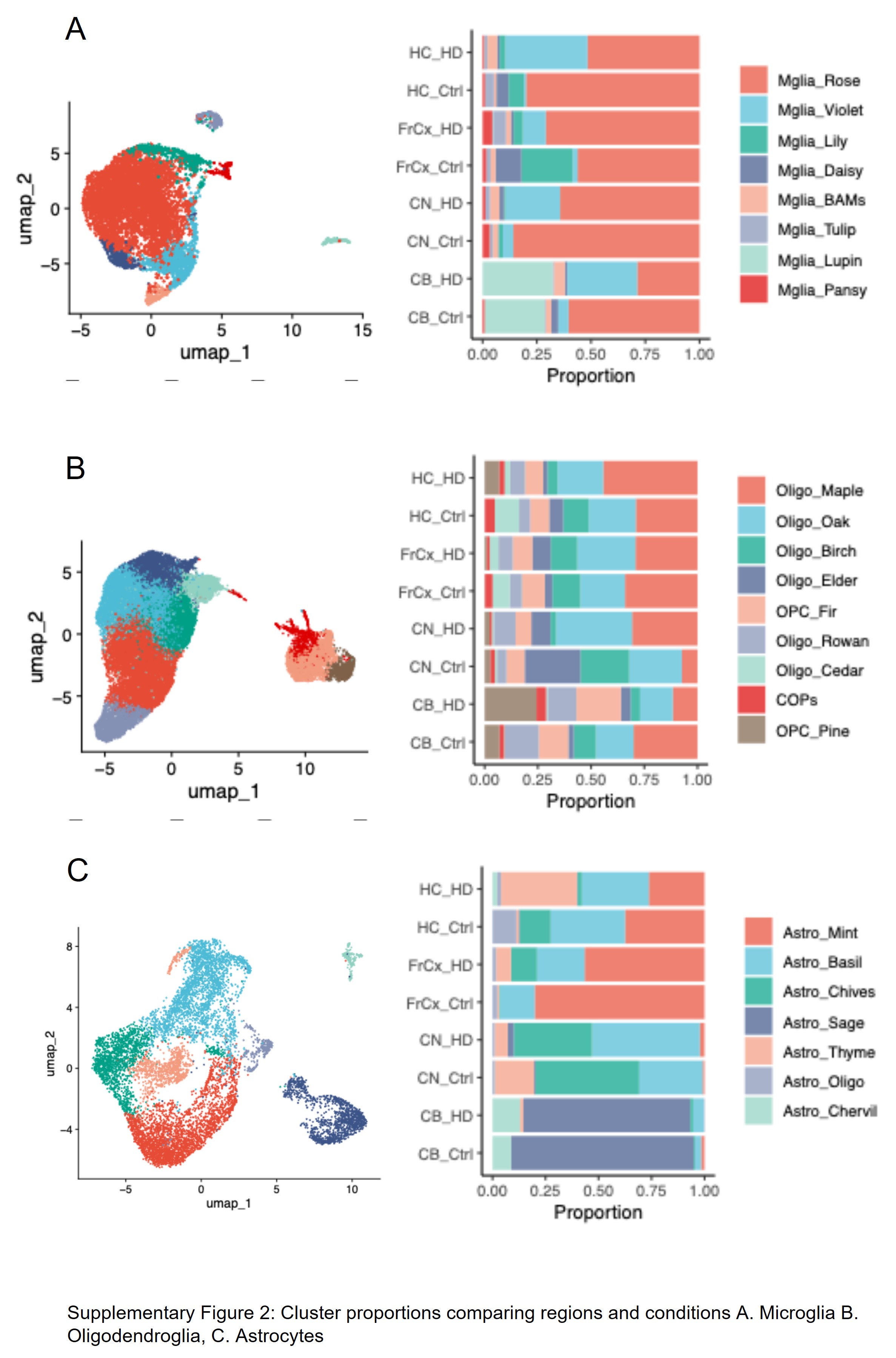

Supplement: Supplementary file 3 — Supplementary Material 3: Figure S3- Tables of top 5 GO terms comparing genes that are different between HD and controls for the different regions for the different cell types. [file 40478_2024_1871_MOESM3_ESM.jpg]
